# Clusterin Protects Hepatocellular Carcinoma Cells from Endoplasmic Reticulum Stress Induced Apoptosis through GRP78

**DOI:** 10.1371/journal.pone.0055981

**Published:** 2013-02-14

**Authors:** Cun Wang, Kai Jiang, Dongmei Gao, Xiaonan Kang, Chun Sun, Qinle Zhang, Yan Li, Lu Sun, Shu Zhang, Kun Guo, Yinkun Liu

**Affiliations:** 1 Liver Cancer Institute, Zhongshan Hospital, Fudan University, Key Laboratory of Carcinogenesis and Cancer Invasion, Ministry of Education, Shanghai, China; 2 Institutes of Biomedical Sciences, Fudan University, Shanghai, China; Complutense University, Spain

## Abstract

Clusterin (CLU) is a stress-activated chaperone, which plays an important role in cancer development and progression through promoting cell survival. However, the exact mechanism of how CLU exerts its cell protective role under ER stress condition is still unclear. Therefore, in order to explore the molecular mechanisms by which CLU inhibited ER stress-induced apoptosis, HCC cell lines were treated with tunicamycin (TN), an ER stress inducer. We found that the expressions of both CLU and GRP78 were increased after TN treatment. Knockdown of CLU expression in SMMC7721 and HCCLM3 cells inhibited GRP78 expression after TN treatment and enhanced ER stress-induced apoptosis, whereas over-expression of CLU in HepG2 cells increased GRP78 expression after TN induction and abolished the effect of TN on cell apoptosis. Furthermore, knockdown of GRP78 expression in CLU-HepG2 cells abrogated the protective role of CLU under ER stress condition. Co-immunoprecipitation (co-IP) and confocal microscopy experiments confirmed the direct interaction between CLU and GRP78 under ER stress condition. The effect of CLU knockdown on GRP78 expression and cell apoptosis in HCC tumors were further determined in orthotopic xenograft tumor model. Knockdown of CLU expression in HCCLM3 cells inhibited GRP78 expression in tumor tissues, accompanied with increased number of apoptotic cancer cells. Moreover, the correlation between CLU and GRP78 expression was further determined in clinical HCC specimens. Taken together, these findings reveal that CLU protects HCC cells from ER stress induced apoptosis at least partially through interacting with GRP78.

## Introduction

The endoplasmic reticulum (ER) is an essential site of cellular homeostasis regulation, especially for the unfolded protein response (UPR). The URP is activated upon the accumulation of misfolded proteins and it is often activated in several liver diseases including hepatocellular carcinoma (HCC) [1]. Accumulating evidence indicated that Glucose-regulated protein 78 (GRP78), a central regulator of UPR, played a critical role in cellular adaptation and survival under stress conditions. Previous study found that GRP78 was overexpressed in a wide range of human tumors including HCC [2], [3], lung cancer [4] and gastric cancer [5], *etc*. Shuda *et al* demonstrated that GRP78 mRNA was elevated in HCC tissues compared to normal liver tissues, which indicated a possible involvement of the ER stress pathway in hepatocarcinogenesis [6]. It has also been reported that GRP78 could mediated the efficacy of several anticancer agents including sorafenib [7], gemcitabine [8] and curcumin [9], which may contribute to the treatment failure in HCC. Given the critical role of GRP78 in cytoprotection and anticancer treatment resistance, further study of the regulatory mechanism for GRP78 will provide novel insights in HCC therapeutics.

Clusterin (CLU), also designated as apolipoprotein J (APOJ), sulfated glycoprotein 2 (SGP2), SP-40 and testosterone-repressed prostate message 2 (TRPM2), is a heterodimeric glycoprotein that influences immune regulation, cell adhesion, transformation, lipid transportation, tissue remodeling, membrane recycling and cell-cell interactions [10]. CLU has several isoforms with distinct functions as a result of alternative splicing and post-translational modifications. sCLU (secretory clusterin) is an ER-targeted, 449-amino acid polypeptide that represents the major product of CLU gene [11]. Another isoform is nuclear CLU (nCLU), which is mainly localizing in the nucleus. Although mature sCLU is processed through the endoplasmic reticulum-Golgi secretory pathway, emerging evidence revealed that sCLU could also localize to cytoplasm [12], [13]. And we focus our study on this isoform of CLU. Up-regulated level of CLU has been reported in HCC [14], [15], breast cancer [16], ovarian cancer [17], colorectal carcinoma [18], and prostate cancer [19]. Several studies confirmed that CLU played an important role in cancer development and progression through promoting cell survival and migration [11]. Its targeted inhibitor (OGX-011) was developed at the University of British Columbia and currently has been used in phase II trials for prostate [20] and lung cancer [21]. The exact mechanism of how CLU exerts its cell protective role is still unclear. Many reports indicated that CLU could inhibit mitochondrial apoptosis through interacting with BAX [13]. In addition, CLU could promote cancer cell survival through activating the Akt and NF-κB pathways [22], [23]. Currently, accumulating evidence suggested that CLU exerted its effects like heat shock proteins under stress condition [10]. Nizard *et al* demonstrated that CLU could suppress ER stress, retro-translocate from ER to the cytosol and inhibit cell apoptosis [12]. Although the elevated expression of CLU under ER stress condition has been confirmed, the molecular mechanisms by which CLU inhibited ER stress-induced apoptosis in HCC remain unclear.

In our present study, we assessed the expressions of CLU and GRP78 in HCC cell lines and explored the regulatory role of CLU on cell apoptosis and GRP78 expression under ER stress condition. We also defined the functional interactions between GRP78 and CLU in HCC cell lines and further determined the correlation between CLU and GRP78 expressions in an orthotopic xenograft tumor model and clinical HCC specimens. Our findings reveal that CLU protects HCC cells from ER stress induced apoptosis through interaction with GRP78.

## Materials and Methods

### 1. Reagents and Antibodies

Tunicamycin (TN) was obtained from Sigma-Aldrich (St. Louis, MO). Goat anti-CLU, FITC-conjugated donkey anti-goat IgG and rhodamine-conjugated donkey anti-rabbit IgG were purchased from Santa Cruz Biotechnology (San Diego, CA). Rabbit anti-GRP78 and PARP antibodies were purchased from Cell Signaling (Danvers, MA, USA). HRP-conjugated anti-goat, mouse, rabbit IgG were purchased from Bio-Rad Laboratories (Hercules, CA). Cell Counting kit and colorimetric TUNEL system were purchased from Dojindo (Kumamoto, Japan) and Promega (Madison, WI), respectively.

### 2. Cell Culture and Western Blot Analysis

Three HCC cell lines (HepG2, SMMC7721 and HCCLM3) were chosen and cultured in our study. HepG2 and SMMC7721 were purchased from the Institute of Biochemistry and Cell Biology, Chinese Academy of Sciences, Shanghai, China. HCCLM3 which is HBV-positive cell line was established at our institute [24]. The cells were cultured in DMEM supplemented with 10% fetal bovine serum. Western blot analysis was performed as previously reported and the densitometry of the bands was analyzed using Quantity One [25].

### 3. Gene Constructs, Lentivirus Production and Transfection

The CLU-RNA interference (RNAi) lentiviral vector was constructed by GeneChem Co, Ltd (Shanghai, China). Two double-stranded oligonucleotides specific targeted to CLU mRNA (shRNA-1-CCGGTGGAGGCATGATGAAGACTCTCTCGAGAGAGTCTTCATCATGCCTCCATTTTTG; shRNA-2-CCGGGATGAAGACTCTGCTGCTGTTCTCGAGAACAGCAGCAGAGT CTTCATCTTTTTG) were annealed and inserted into the shRNA expression vector pGCSIL-GFP. The cDNA encoding CLU was amplified by reverse transcription polymerase chain reaction (RT-PCR) and cloned into pGC-FU-GFP vector, which generated GFP-tagged-CLU. The lentivirus was generated and harvested as described previously (Shanghai GeneChem Co., Ltd., Shanghai, China). Then the lentivirus was transfected into targeted cells with a multiplicity of infection (MOI) of 10 to 50 (optimal MOI is 20).

We silenced GRP78 expression in HepG2 cells with small interfering RNA (siRNA). The siRNA sequences against GRP78 were 5′-CCACCAAGAUGCUGACAUU-3′ (sense); 5′-AAUGUCAGCAUCUUGGUGG-3′ (antisense) and 5′-GAGGCUUAUUUGGGAAAGA-3′ (sense); 5′-UCUUUCCCAAAUAAGCCUC-3′ (antisense). The transfection protocol has been described previously [26].

### 4. CCK8

CCK8 assay was performed to determine cell viability after TN treatment with indicated concentrations. The procedure was performed as previously describe [25].

### 5. Apoptosis Measurement by Flow Cytometry and PARP Detection

The following two methods were used to assess TN-induced apoptotic cell death: the measurement of apoptotic cells by flow cytometry (sub-G1) and Western blot analysis of PARP cleavage. The procedure was performed as previously describe [27].

### 6. Immunocytochemistry and Confocal Microscopy

Cells were grown on glass coverslips. After an attachment period of 24 h, cells were incubated with vehicle or TN (1 µg/ml) for 24 h. Then cells were fixed in 4% formaldehyde at room temperature for 30 min and permeabilized with 0.1% Triton-X in PBS for 10 min and blocked with 10% donkey serum in PBS for 1 h. The coverslips were then incubated with rabbit anti-GRP78 antibody for 1 h, followed by rhodamine-conjugated donkey anti-rabbit IgG for 30 min. Subsequently, goat anti-CLU antibody was applied for another hour, and then stained with FITC-conjugated donkey anti-goat IgG for 30 min. Finally, cells were washed and stained with DAPI. The signals were detected with A1R MP Multiphoton Confocal Microscope and analyzed with NIS-elements microscope imaging software (Nikon Instruments Inc, Melville, NY, USA).

### 7. Co-Immunoprecipitation (co-IP)

For co-IP, pre-cleared protein from whole cell lysates were incubated with goat anti-CLU antibody which is conjugated to AminoLink Plus Resin (Pierce) overnight at 4°C. The co-IP targets were disassociated from the immobilized antibodies on the AminoLink Plus Resin by the gentle (non-reducing, non-denaturing) elution buffer. Eluted proteins were resolved using 10% SDS-PAGE, followed by Western blot with appropriate antibodies and detection using ECL (GE, Healthcare, Piscataway, NJ).

### 8. Orthotopic Xenograft Tumor Model

The *in vivo* experiments were carried out strictly in accordance with a protocol approved by the Shanghai Medical Experimental Animal Care Committee (Permit Number: 2009-0082). HCCLM3 cells infected with lentivirus (CLU-shRNA HCCLM3 and mock cells) were implanted subcutaneously into the upper left flank region of nude mice. When the tumor reached 1 cm in diameter, they were cut into 2×2×2 mm^3^ sized pieces, and implanted into livers of 12 nude mice. After 6 weeks, the mice were sacrificed and the tumors were fixed with 10% buffered formalin and embedded with paraffin for further study.

### 9. Immunohistochemistry Analysis on Tissue Microarrays

All samples were obtained from the Department of Hepatobiliary Surgery, First Affiliated.

Hospital of Guangxi Medical University (Nanning, China). HCC diagnosis was based on World Health Organization criteria. Ethical approval was obtained from the Research Ethics Committee of First Affiliated Hospital of Guangxi Medical University, and written informed consent was obtained from each patient. Tissue microarrays were constructed using formalin-fixed, paraffin-embedded tissue samples derived from 96 HCC patients. Immunohistochemical stains were performed using anti-CLU (1∶200 dilution) and anti-GRP78 (1∶100 dilution) as previously described. Then the optical density of the images was analyzed by IPP software (image-pro plus 5.1).

### 10. *In situ* Apoptosis Detection by TUNEL Staining

Paraffin-embedded, 5 µm thick sections were used to identify apoptotic cells by using TUNEL assay kit according to the manufacturer’s instructions. The extent of apoptosis was evaluated by counting the TUNEL-positive cells (brown-stained).

### 11. Statistical Analysis

Statistical analysis was performed with SPSS 15.0 for Windows (SPSS, Chicago, IL). Quantitative variables were analyzed by Student t tests. *P*<0.05 was considered statistically significant.

## Results

### 1. TN Induces the Expression of CLU and GRP78 in HCC Cells

**Figure 1:**
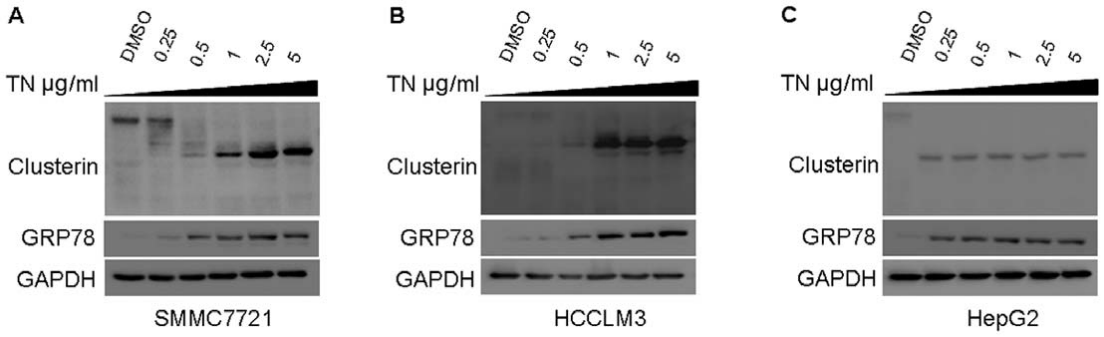
TN induces the expressions of CLU and GRP78 in HCC cell lines *in vitro*. (A–C) SMMC7721, HCCLM3 and HepG2 cells were treated for 24 h with TN for the indicated doses. Protein extracts were analyzed for CLU, GRP78 and GAPDH. TN treatment induced both CLU and GRP78 expressions and the molecular weight of CLU was decreased after TN treatment.

The effects of TN on the expression of CLU and GRP78 levels were evaluated in SMMC7721, HepG2 and HCCLM3 cells. TN treatment increased both CLU and GRP78 expressions in the three HCC cell lines (Figure 1). When cells were treated with the ER stress inducer TN, the molecular weight of CLU was obviously decreased and we speculated that TN which is also known as an N-glycosylation inhibitor might alter the glycosylation of CLU (Figure 1).

### 2. Knockdown of CLU Enhances ER Stress Induced Apoptosis which may be Associated with Down-regulation of GRP78

**Figure 2:**
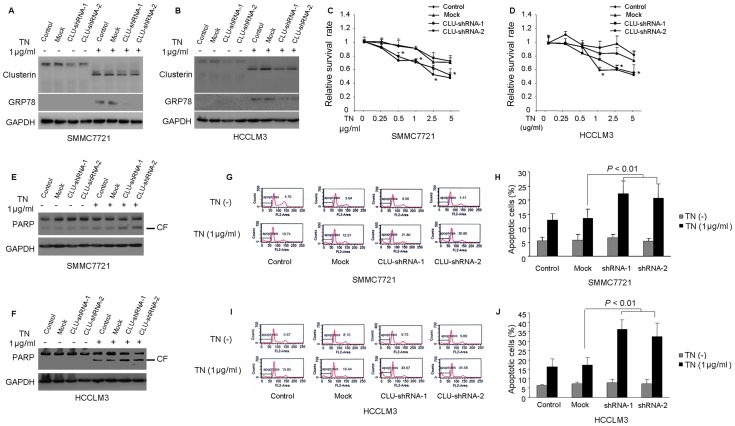
CLU knockdown enhances TN treatment induced apoptosis in SMMC7721 and HCCLM3 cells. (A–B) SMMC7721 and HCCLM3 cells were infected twice for a total of 2 days (1 day for each infection). The expression of CLU in SMMC7721 and HCCLM3 cells were inhibited through lentivirus-mediated shRNA interfering followed by 1 µg/ml of TN treatment for 24 h. Protein extracts were analyzed for CLU, GRP78 and GAPDH. (C–D) CCK8 assay was performed to determine cell viability after TN treatment with indicated concentrations for 24 h. The results indicated a synergistic effect of CLU knockdown with TN treatment. The values of relative survival rate were normalized with untreated cells. *P<0.05, versus mock group. TN-induced cell apoptosis in SMMC7721 and HCCLM3 cells were measured by Western blot analysis of PARP cleavage (CF, cleaved form) (E–F) and flow cytometry (sub-G1) (G–J). All these data were from a representative experiment and the representative result was from at least three independent experiments.

In our previous study we found that the expression of CLU is quite high in SMMC7721 and HCCLM3 cell lines than HepG2 [28], so we employed lentivirus-mediated shRNA to knockdown the expression of CLU in SMMC7721 and HCCLM3 cells. And then we used lentiviral infection to introduce a CLU cDNA expression vector into HepG2 cells to further confirm the role of CLU. Three independent experiments indicated that compared with mock group, treatment with specific shRNA-1 and shRNA-2 resulted in about 63.3±8.2% and 55.0±4.4% silencing of CLU in SMMC7721 cells and 74.6±6.0% and 70.3±9.4% silencing of CLU in HCCLM3 cells (*P*<0.05, Figure 2A and B). SMMC7721 and HCCLM3 cells were also treated with 1 ug/ml TN for 24 h and the expressions of CLU and GRP78 were further measured. Results showed that the inducing effects of TN on CLU were obviously inhibited in CLU-shRNA cells compared with mock cells (Figure 2A and B). The actual inhibitive percentage of shRNA-1 and shRNA-2 was about 53.3±9.5% and 59.7±7.5% in SMMC7721 cell line and 61.0±9.4% and 62.3±6.6% in HCCLM3 cells line (*P*<0.05, compared with mock group). Notably, as shown in Figure 2A and B, compared with mock cells, a parallel down-regulation of GRP78 was also shown in CLU-shRNA cells after TN treatment.

CCK8 assays demonstrated that the down-regulation of CLU potentiated TN-induced cell apoptosis, starting at the concentration of 0.5 µg/ml in SMMC7721 cells and 1 µg/ml in HCCLM3 cells (Figure 2C and 2D).

Additionally, we studied cell apoptosis in these cells after 1 µg/ml TN treatment for 24 h. Down-regulation of CLU in SMMC7721 and HCCLM3 cells did not show any effect on cell viability in normal culture condition. However, the down-regulation of CLU sensitized those cells to TN-induced apoptosis, which were detected by PARP cleavage (Figure 2E and 2F) and flow cytometry analysis (Figure 2G–J). These results indicated that knockdown of CLU might reduce the adaptive ability of HCC cells to TN treatment.

### 3. CLU Protects HCC Cells from ER Stress Induced Apoptosis through Up-regulation of GRP78

**Figure 3:**
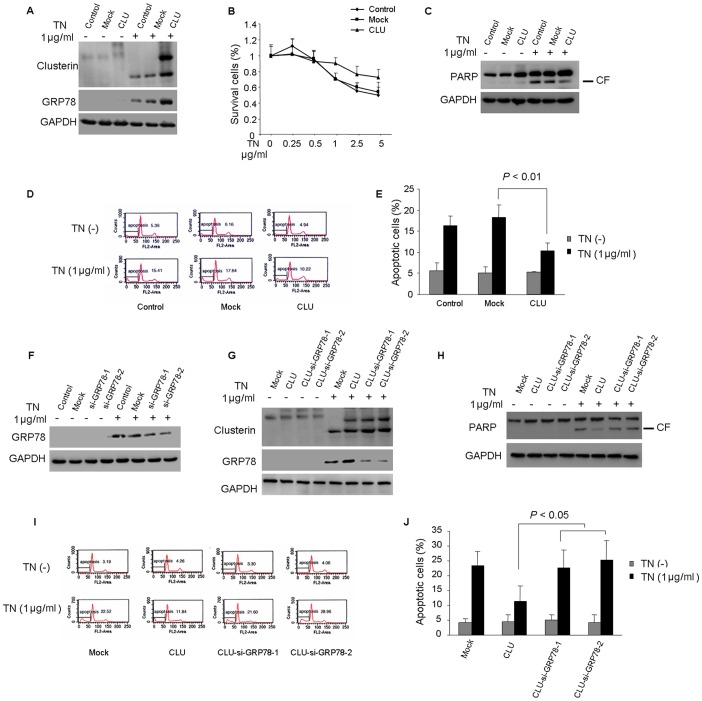
CLU induction following TN treatment is cytoprotective via increasing the expression of GRP78. (A) Control, mock and CLU-HepG2 cells were treated with 1 µg/ml of TN for 24 h. Protein extracts were analyzed for CLU, GRP78 and GAPDH. (B) CCK8 assay was performed to determine cell viability after TN treatment with indicated concentrations for 24 h. The values of relative survival rate were normalized with untreated cells. *P<0.05, versus mock group. (C–E) TN-induced cell apoptosis in HepG2 cells were measured by Western blot analysis of PARP cleavage (CF, cleaved form) and flow cytometry (sub-G1). The results indicated that CLU played a protective role on HCC cells under ER stress condition. (F) HepG2 cells were transfected with scramble siRNA or siRNA specific for GRP78 followed by 1 µg/ml of TN treatment for 24 h. Protein extracts were analyzed for GRP78 and GAPDH. Knockdown of GRP78 expression in HepG2 inhibited TN induced GRP78 expression. (G) CLU overexpressed HepG2 cells were transfected with scramble siRNA or siRNA specific for GRP78 followed by 1 µg/ml of TN treatment for 24 h. Protein extracts were analyzed for CLU, GRP78 and GAPDH. (H–J) TN-induced cell apoptosis in HepG2 cells were measured by Western blot analysis of PARP cleavage (CF, cleaved form) and flow cytometry (sub-G1). The results suggested GRP78 may be important in mediating the protective effect of CLU under ER stress condition. All these data were from a representative experiment and the representative result was from at least three independent experiments.

In order to further confirm the protective role of CLU under ER stress condition, we generated a HepG2 cell line over-expressing CLU which is tagged with GFP. After TN treatment, over-expression of CLU in HepG2 cells significantly increased both CLU and GRP78 expressions with 8.4±1.4 fold and 4.2±1.1 fold up-regulation, respectively, compared with mock cells (*P*<0.05, Figure 3A).

Our data also showed that over-expression of CLU in HepG2 cells abolished the effect of TN on apoptosis, starting at the concentration of 1 µg/ml (Figure 3B). In concordance with the study in knockdown cells, ectopically expressed CLU in HepG2 cells did not show any effects on cell viability under normal culture condition. However, our data showed that up-regulation of CLU diminished PARP cleavage in HepG2 cells under ER stress condition (Figure 3C) and partly abolished the effect of TN on apoptosis with statistical significance (*P*<0.01, Figure 3D and E). These results further indicated that CLU played a key role in mediating the apoptotic effect of TN on HCC cells.

In addition, in order to determine whether the knockdown of GRP78 could counteract the protective effects of CLU under ER stress conditions, HepG2 cells were transfected with siRNA specific to GRP78. We found that after treatment with specific GRP78 siRNA, TN induced GRP78 up-regulation was significantly suppressed, with about 2-fold decrease compared with mock cells (*P*<0.05, Figure 3F). Next, the CLU-HepG2 cells were transfected with siRNA specific to GRP78. As shown in Figure 3G, TN induced GRP78 expression was suppressed by GRP78 siRNA with about 4-fold decrease compared with CLU-HepG2 cells (*P*<0.05). In addition, down-regulation of GRP78 counteracted the protective effects of CLU, which was associated with PARP cleavage (Figure 3H), and increased apoptotic cell death in CLU-HepG2 cells (Figure 3I and J). These results suggested GRP78 might be important in mediating the protective effect of CLU under ER stress condition.

### 4. CLU Associates with GRP78 under ER Stress Condition

**Figure 4:**
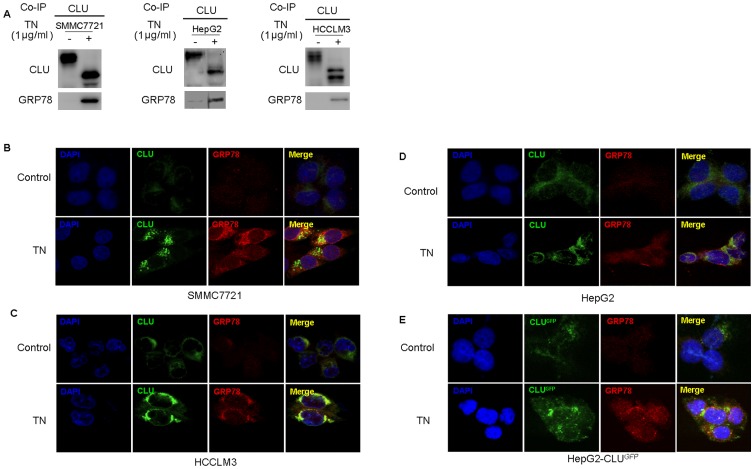
CLU associates with GRP78 under ER stress conditions. (A) Western blot confirmed the presence of GRP78 in CLU co-IP. HCC cell lines (SMMC7721, HCCLM3 and HepG2) were treated with 1 µg/ml TN for 24 h. Pre-cleared protein from whole cell lysates were incubated with goat anti-CLU antibody and then detected with anti-GRP78. (B–D) HCC cell lines (SMMC7721, HCCLM3 and HepG2) were treated with 1 µg/ml TN for 24 h and then co-immunostained with anti-CLU (green) and anti-GRP78 (red). The signals were detected with A1R MP Multiphoton Confocal Microscope. (E) CLU-HepG2 cells were treated with 1 µg/ml of TN for 24 h, the GRP78 was detected by immunostaining with anti-GRP78 (red). Ectopically expressed CLU is tagged with GFP (green). All these data were from a representative experiment and the representative result was from at least three independent experiments.

To better define the relationship between CLU and GRP78, CLU was immunoprecipitated from three HCC cell lines (SMMC7721, HCCLM3 and HepG2) with or without TN induction and Western blotting was performed using CLU and GRP78 antibodies, respectively. We found that GRP78 was highly induced by TN treatment and was predominantly associated with CLU in the co-IP (Figure 4A). Confocal microscopy confirmed the co-localization of CLU and GRP78 in cytoplasm in all three cell lines after TN treatment (Figure 4B–D). Interestingly, the ectopically expressed CLU, which is tagged with GFP, was also co-localized with GRP78 in HepG2 cells (Figure 4E). These results revealed the direct interactions between GRP78 and CLU under ER stress conditions.

### 5. Silencing of CLU Expression Induces GRP78 Down-regulation and Cell Apoptosis *in vivo*

**Figure 5:**
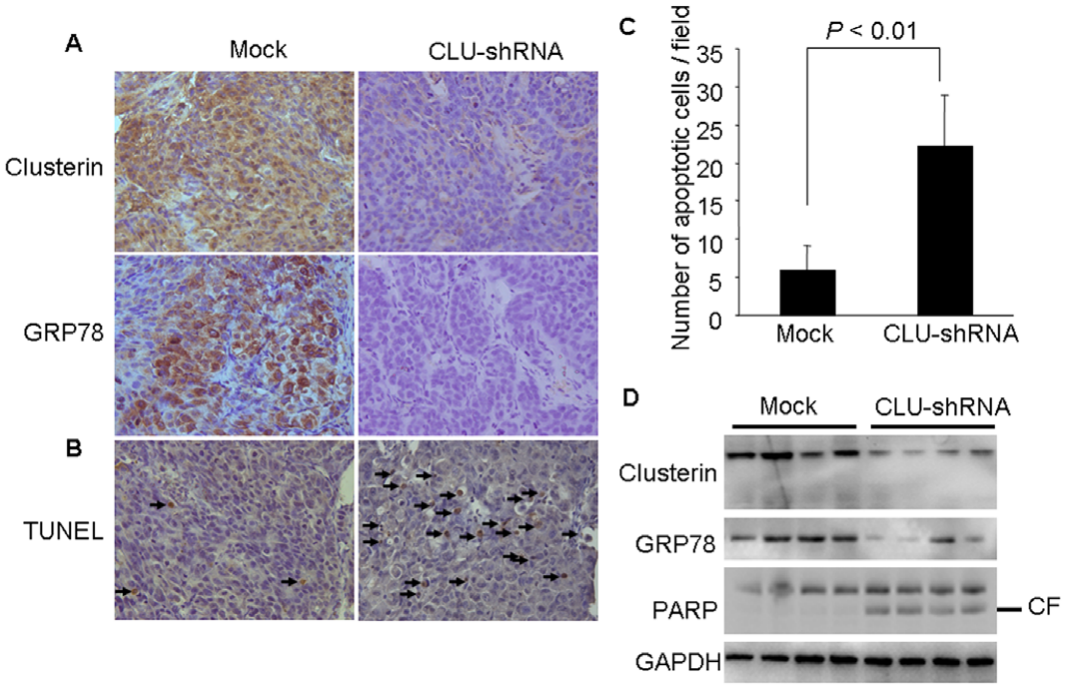
Silencing of CLU induces GRP78 down-regulation and cell apoptosis in *vivo.* (A) Immunohistochemistry staining with anti-CLU and anti-GRP78 were performed on serial sections of tumor specimen (×400). (B–C) The apoptotic cells were identified by TUNEL staining. The number of TUNEL-positive cells in CLU-shRNA group was significantly elevated compared with mock group (× 400). (D) Total proteins were extracted from the xenograft tumors and CLU, GRP78 and PARP cleavage (CF, cleaved form) were analyzed by Western blotting.

To correlate biological response and the mechanism identified in vitro, the effects of CLU knockdown on GRP78 and cell apoptosis in orthotopic xenograft tumor tissues were examined by immunohistochemical staining and Western blot analysis. Immunohistochemical staining with a CLU antibody confirmed the expression of CLU in CLU-shRNA HCCLM3 and mock cells derived tumors. The expression of CLU was significantly inhibited in CLU-shRNA group compared with mock group (Figure 5A). This was also accompanied by a corresponding down-regulation of GRP78 in CLU-shRNA group (Figure 5A). Then the expressions of CLU and GRP78 in four representative tumors in each group were assessed by Western blotting. As shown in Figure 5D, the Western blot results corroborated the immunohistochemical staining ones. In addition, the effect of CLU on tumor cell apoptosis *in vivo* was examined through TUNEL staining (Figure 5B and C) and cleavage of PARP (Figure 5D). The number of apoptotic cells was significantly increased from 6/field in mock group to 22/field in CLU-shRNA group (*P*<0.01). Furthermore, we also found that down-regulation of CLU and GRP78 was correlated with cleavage of PARP in tumor tissues (Figure 5D).

### 6. Correlation between CLU and GRP78 Expression in Clinical HCC Specimens

**Figure 6:**
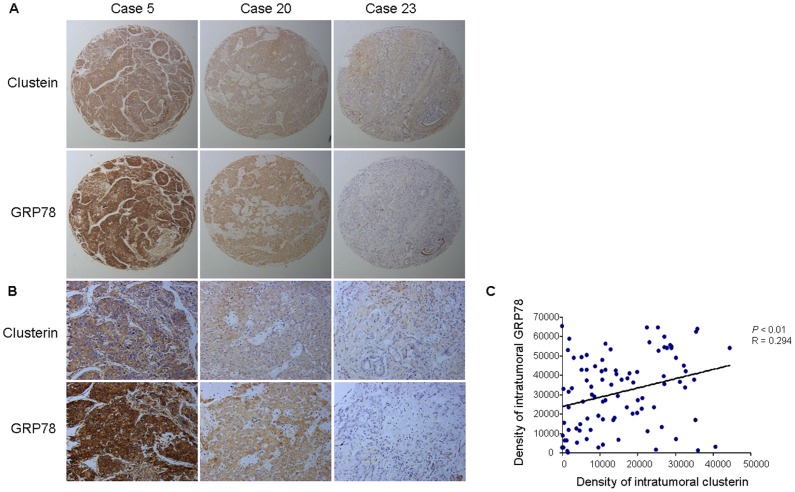
There was a correlation between the levels of CLU and GRP78 in clinical HCC specimens. (A–B) Representative immunostaining for CLU and GRP78 is shown for three patient samples (A: ×50; B: ×200). (C) Positive correlation between CLU expression and GRP78 level was examined in tumor tissues derived from 96 patients (r = 0.294, *P*<0.01).

To further investigate the correlation between the expression of CLU and GRP78, levels of CLU and GRP78 were compared in 96 human HCC tissues using a tissue microarray (TMA). Our results revealed a correlation between CLU expression and the level of GRP78 (Pearson’s correlation, r = 0.294, *P*<0.01) (Figure 6A and B). Three representative immunostaining results for CLU and GRP78 were shown for three patient samples (Figure 6A).

## Discussion

Clusterin (CLU) is a chaperone that inhibits protein aggregation and precipitation, otherwise induced by physical or chemical stresses. CLU is a protective molecule by helping cells to cope with stress condition in cancer cells. Many studies demonstrated that CLU conferred resistance to anti-cancer agents in many kinds of cancer [29], [30], [31], [32]. Emerging clinical evidences as well as preclinical findings revealed that OGX-011, a CLU inhibitor, could improve the efficacy of chemotherapy through sensitizing cancer cells to drug induced apoptosis [33], [34]. Several anti-cancer agents could activate ER stress, thus increasing CLU expression level, which may cause an acquired treatment-resistant phenotype. We have previously shown that CLU facilitated HCC metastasis through inducing epithelial-mesenchymal transition [28]; however the role of CLU in regulating ER stress-induced apoptosis has not been clearly identified.

In this study, we hypothesized that CLU might have crucial effects on ER stress induced apoptosis. After treatment with TN, an inducer of ER stress, the level of CLU was elevated in HCC cell lines, accompanied with the corresponding up-regulation of GRP78. GRP78, an important ER-resident chaperon, could also mediate the efficacy of several anticancer agents. Then we focused our study on the regulatory relationship between CLU and GRP78. Knockdown of CLU in SMMC7721 and HCCLM3 cells suppressed GRP78 induction after TN treatment and increased ER stress-induced apoptosis, whereas over-expression of CLU in HepG2 cells increased GRP78 expression after TN induction and abolished the effect of TN on cell apoptosis. Knockdown of GRP78 abrogated the protective role of CLU in HepG2 cells under ER stress condition. Li et al previously demonstrated that GRP78 regulated CLU stability and retrotranslocation in prostate cancer [35]. However, we found that the expression of CLU did not change in HepG2 cells after GRP78 down-regulated, even at ER stress condition. The difference may be derived from distinctive regulatory mechanism in different types of cancer. Our results indicated that CLU played an important role in cytoprotection under ER stress condition at least partially through its regulatory effects on GRP78, but not vice versa. However, the precise regulatory mechanism of CLU on GRP78’s expression needs to be further investigated.

CLU is a conserved protein mediating cytoprotective role through its chaperone-like activity which is similar to heat shock proteins [10]. GRP78 is an essential modulator of URP via its protein binding activities. Given the direct regulatory role of CLU on GRP78, if there is a direct interaction between them attracted our full attention. Co-IP and confocal microscopy indicated that CLU could directly interact with GRP78 in HCC cell lines under ER stress condition. The ectopically expressed CLU, which is tagged with GFP, was also co-localized with GRP78 after treatment with TN. On the basis of these evidences, we concluded that CLU could regulate the expression of GRP78 through the direct interaction. In our study, the antibody against CLU is an affinity purified goat polyclonal antibody raised against a peptide mapping at the C-terminus of sCLU α-chain. So we can conclude the direct interaction between GRP78 and cytoplasmic sCLU. nCLU lacking the leader peptide would not enter the endoplasmic reticulum, mainly localizing in the nucleus. GRP78 is mainly residing in the ER. So we speculated that only cytoplasmic sCLU could interaction with GRP78.

Several studies have correlated the overexpression of CLU with HCC [14]. However, mechanisms of how CLU influences cell apoptosis in HCC are still unclear. One novel finding in our study was that CLU was closely associated with cell apoptosis in the orthotopic xenograft model. Similar to the results obtained by *in vitro* experiments, knockdown of CLU in HCCLM3 cells inhibited GRP78 expression in tumor tissues, accompanied with increased number of apoptotic cancer cells. Finally we confirmed the correlation between GRP78 expression and CLU levels in tumor tissues derived from 96 HCC patients. To our knowledge, it is the first time to explore the correlation between GRP78 expression and the level of CLU in cancer tissues. All these results further confirmed that CLU and GRP78 might be candidate targets for HCC therapy.

### Conclusion

We concluded that CLU could protect HCC cells from ER stress-induced apoptosis by regulating GRP78. And our findings defined the interaction between CLU and GRP78 under ER stress condition. In addition, we confirmed the correlation between CLU and GRP78 expression levels in orthotopic xenograft tumor tissues and clinical HCC specimens.
